# Asymmetry in Species Regional Dispersal Ability and the Neutral Theory

**DOI:** 10.1371/journal.pone.0024128

**Published:** 2011-08-25

**Authors:** Jiajia Liu, Shurong Zhou

**Affiliations:** Key Laboratory of Arid and Grassland Ecology under the Ministry of Education, School of Life Sciences, Lanzhou University, Lanzhou, China; University Copenhagen, Denmark

## Abstract

The neutral assumption that individuals of either the same or different species share exactly the same birth, death, migration, and speciation probabilities is fundamental yet controversial to the neutral theory. Several theoretical studies have demonstrated that a slight difference in species per capita birth or death rates can have a profound consequence on species coexistence and community structure. Whether asymmetry in migration, a vital demographic parameter in the neutral model, plays an important role in community assembly still remains unknown. In this paper, we relaxed the ecological equivalence assumption of the neutral model by introducing differences into species regional dispersal ability. We investigated the effect of asymmetric dispersal on the neutral local community structure. We found that per capita asymmetric dispersal among species could reduce species richness of the local community and result in deviations of species abundance distributions from those predicted by the neutral model. But the effect was moderate compared with that of asymmetries in birth or death rates, unless very large asymmetries in dispersal were assumed. A large difference in species dispersal ability, if there is, can overwhelm the role of random drift and make local community dynamics deterministic. In this case, species with higher regional dispersal abilities tended to dominate in the local community. However, the species abundance distribution of the local community under asymmetric dispersal could be well fitted by the neutral model, but the neutral model generally underestimated the fundamental biodiversity number but overestimated the migration rate in such communities.

## Introduction

The unified neutral theory, an intriguing and yet controversial explanation for species diversity patterns, has attracted much attention and has stimulated much new thinking about diversity [1]–[16]. According to the neutral model[8], [9], species abundance distribution in a local community can be generated by simply specifying the community size, birth, death, and migration rates, presuming that species are ecologically identical in terms of their per capita contribution to species diversity (neutral assumption). A dynamical equilibrium of species diversity can be maintained via the balance between extinction and immigration from a metacommunity. It is remarkable that under this simple and counterintuitive hypothesis, the neutral theory can predict species abundance distributions that are similar to those in some real communities [7], [9], [13], [17], [18].

The assumption of ecological equivalence or ecological symmetry is fundamental to the neutral theory, but it is also one of the main causes of the controversy. In real communities, species can be quite different from each other in their life history traits that contribute to their demographic rates. Some theoretical studies have examined the robustness of the neutral model against the differences in species fecundity or mortality rates [16], [19], [20]. Slight differences in species per capita fecundity result in a dramatic decline in species coexistence time and in significant departures of species abundance distributions from those predicted in neutral cases [16], [20]. Species with a higher per capita fecundity factor dominate the competition and thus have higher relative abundances [16]. In another study, Yu et al. [19] relaxed the assumption of ecological equivalence by allowing mortality rates to differ across species and found a considerable drop in persistence times which are not plausible for speciation to occur. In this case, community composition again becomes highly deterministic, with species of high mortality rates more likely to become extinct than those of low mortality rates. These studies all come to the conclusion that the neutral model is fragile with regards to the ecological equivalence assumption, and the effects of species differences in either fecundity or mortality rates can be substantial and can lead to a deterministic rather than stochastic outcome of competition and community assembly.

However, Hubbell suggested that the ecological equivalence assumption works well as a first approximation [9], [10], [17]. Based on the theoretical work by Hurtt and Pacala [21], Hubbell argued that dispersal and recruitment limitation could delay competitive exclusion, essentially without any limit, by reducing the effect of competitive asymmetries among the species. But this statement seems questionable. On the one hand, Tang and Zhou [22] recently argued that the effects of niche differentiation and recruitment limitation were blended together in Hurtt and Pacala's model because of the assumption of niche differentiation in space among species. By removing niche differentiation, they showed that even a slight competitive asymmetry among species requires an extremely strong dispersal and recruitment limitation to work against it [22]. On the other hand, dispersal is assumed to be symmetric among individuals and species in both the neutral model and Hurtt and Pacala's model. Some other studies have also addressed the migration parameter of the neutral model, but the per capita dispersal rates were all assumed to be the same among species [3], [23], [24]. In real communities, however, dispersal is rarely equivalent among different species. The number of immigrants entering a local community per time interval is a stochastic variable. The relative abundance of each species in the migration pool may also fluctuate at random, even if all of the species share the same average migration probability. Most importantly, species may differ in migration probability not only because of stochasticity such as the random sampling process, but also the dispersal ability itself varies considerably across species [25].

To our knowledge, only one theoretical study tested the robustness of the neutral model against variance in migration rate [26]. Hu et al. extended the local neutral community model by introducing stochasticity into the immigration rate [26]. Two modes of stochastic variation in the immigration rate were considered. One is the temporal variation in the total number of immigrants per unit time, and the other is the temporal variation in the relative abundance of any given species in the immigration pool due to the sampling effect. They demonstrated that local species diversity is a function not only of the mean but also of the variance in the immigration rate. It is expected that rare species are more sensitive to changes in migration than abundant species. Hence, variance in the immigration rate acts to reduce the number and abundance of rare species and to favor the common species in local communities. In the end, variance in species migration rates reduces species richness in local communities. However, the effects of species differences in dispersal ability on shaping the local community structure still remain unknown. Most importantly, investigations on relationship between species abundance and its regional dispersal ability are needed.

In this paper, we considered a local community embedded within a metacommunity. We relaxed the ecological equivalence assumption of the neutral model by introducing differences into species' natural dispersal ability from the metacommunity to the local community. Diversity of such a local community was maintained by the balance among local birth, death and immigration from the outer metacommunity. The local community was neutral. Asymmetry only existed in the input of individuals and species by immigration from the corresponding metacommunity. We investigated by simulation the relative importance of dispersal asymmetry among species versus the ecological drift in shaping local community structures. We also recorded the relationship between species per capita dispersal ability from the metacommunity to the local community and species relative abundance in the local community. Finally, we evaluated the ability of the neutral model in fitting species abundance distributions of local communities with dispersal asymmetries.

## Results

**Figure 1:**
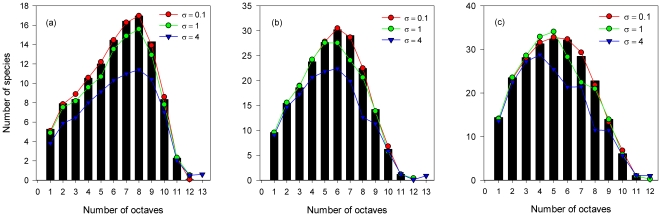
Effects of species differences in migration probability from the metacommunity to the local community on the local community structure. Parameter values: *θ* = 50, and (a) *m* = 0.01, σ = 0, 0.1, 1 and 4, respectively; (b) *m* = 0. 1, σ = 0, 0.1, 1 and 4, respectively; (c) *m* = 0.3, σ = 0, 0.1, 1 and 4, respectively. Large differences in species per capita immigration ability result in decreased species richness and deviation of species abundance distributions in local communities from those predicted by the neutral theory. The black bars are for the neutral model. The results are the average over 100 replicate simulations, and the variances are similar for different values of σ, which are not shown in the figure for clarity.

Fig. 1 illustrates the influence of interspecific differences in dispersal rates on the distributions of species abundance in local communities. As expected, the species abundance distributions are close to the predictions by the pure neutral theory (σ = 0) when differences among species migration rate are small, i.e. σ = 0.1. Medium intensity of dispersal asymmetries (the standard deviation equals the mean, i.e. σ = 1) also results in species abundance distributions similar to the neutral predictions. When the standard deviation becomes even larger, species richness decreases dramatically and the distribution of relative abundances deviates far from the patterns predicted in the neutral cases with a fixed *m* and no deviation (σ = 0). In this case, there appear a few species with very high abundances, whereas the number of species with rare and medium abundances decreases.

**Figure 2:**
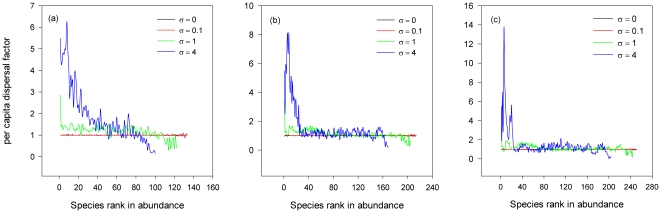
The relationship between species per capita regional migration probability and species rank in abundance in local communities. Parameter values are the same as in Fig.1.

Contrary to the neutral model, there is a positive correlation between species per capita dispersal factor and species rank in abundance for especially common species (Fig. 2). With the difference in species dispersal ability, the local community structure becomes highly deterministic rather than random as in the neutral model. Those species with higher dispersal abilities can rescue themselves from extinction in the local community and increase their relative abundance, while rare species are those with relatively low dispersal rates from the metacommunity. In other words, a large difference in species dispersal ability, if there is, can overwhelm the effect of the stochastic drift and play a dominant role in the community assembly.

**Figure 3:**
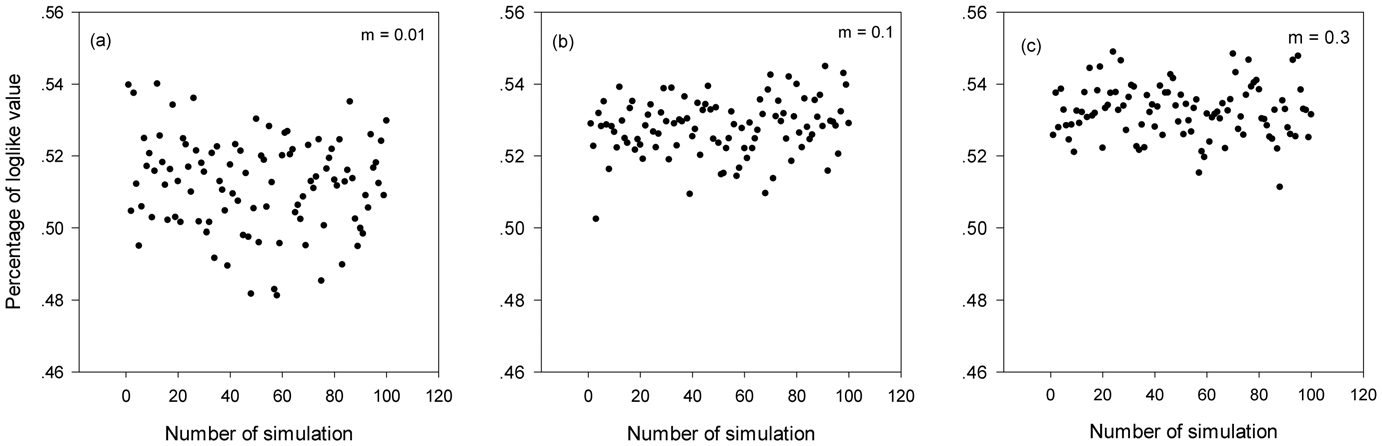
Goodness of fit of the neutral model to relative abundance distributions in local communities with differences in species' per capita immigration probability. Parameter values: (a) *m* = 0.01, σ = 4; (b) *m* = 0.1, σ = 4; (c) *m* = 0.3, σ = 4. Each point represents the percentage value by comparing the LV and LV*_i_* (*i* = 1, …, 100) for each replicate simulation of the same parameter set.

Besides the effects of dispersal asymmetries on community structures described above, the neutral model fits the local neutral communities with dispersal asymmetries very well (Fig. 3). For example, all the percentages of log-likelihood values are around 0.5 when the standard deviations are even 4 times as large as the mean per capita dispersal factors. This demonstrates the strong ability of the neutral model in predicting species abundance distributions in even neutral communities with asymmetric immigrations. However, the estimated parameter values for the neutral model are quite different from the real ones in communities with asymmetric immigrations (Table 1). The neutral theory generally predicts lower fundamental biodiversity parameters (*θ*) for the local communities with large differences in species regional dispersal abilities. The larger the variance in species dispersal abilities, the greater the underestimates of the fundamental biodiversity number will be. The neutral theory also overestimates the migration rate when species differ largely in their dispersal abilities.

**Table 1:** Maximum likelihood estimates (Etienne 2005) of *θ* and *m* by the neutral model, with *J*  = 10 000, *θ* = 50.

Parameters used in simulations			Parameters estimated by the neutral model	
*θ*	*m*	*σ*	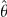 ( 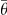 )	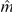 ( 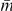 )
50	0.01	0.1	59.2 (44.6)	0.008 (0.046)
		1	33.6 (37.6)	0.021 (0.062)
		4	18.1 (18.9)	0.083 (0.149)
	0.1	0.1	51.9 (52.9)	0.087 (0.100)
		1	42.9 (42.9)	0.154 (0.177)
		4	30.3 (30.2)	0.327 (0.375)
	0.3	0.1	51.5 (51.6)	0.269 (0.282)
		1	46.5 (46.5)	0.361 (0.395)
		4	35.1 (35.0)	0.657 (0.678)

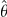
 and 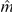 are estimated values of θ and m from the mean species abundance distribution averaged over 100 replicate simulations, whereas 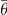 and 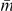 in the brackets are averages over estimated values for 100 replicate simulations for each parameter set.

## Discussion

The ecological equivalence assumption is fundamental to the neutral theory. However, the real communities are unlikely to be “neutral”. Hence, the ecological equivalence assumption of the neutral model has been frequently criticized from both theoretical and empirical perspectives. The strict assumption of equivalence among individuals finds little empirical support in real communities [27], [28]. Theoretical studies have shown that slight deviations from species symmetries in fecundity or mortality rates can significantly violate the predictions of the neutral model [16], [19], [20]. In this paper, we showed that dispersal asymmetry can also result in departures of species abundance distributions in local communities from the neutral predictions with the same mean migration rates (Fig. 1). Although the local communities with regional asymmetric dispersal abilities can be fitted by the neutral model, the neutral model generally predicted lower fundamental biodiversity numbers and higher migration rates than those used in the simulations (Table 1). This phenomenon can be understood as follows. In the neutral case, the relative abundance of species *i* is *p_i_* = *P_i_*/*J_m_* in the metacommunity. The probability that an immigrant to the local community belongs to species *i* is proportional to *p_i_*. Let us denote it as a *p_i_*-metacommunity. With dispersal asymmetry introduced in the model, an immigrant is from species *i* with probability 
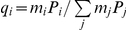
. In this case, selecting an immigrant from a metacommunity with regional dispersal asymmetry actually resembles randomly sampling immigrants from a metacommunity in which the relative abundance of species *i* is *q_i_* (*q_i_*–metacommunity). But both the diversity and evenness of such a *q_i_* –metacommunity decrease with increasing σ (a *q_i_*–metacommunity with σ = 0 is actually a *p_i_*-metacommunity). The Simpson diversity index is 0.98 and *θ* is 50 for σ = 0. The average Simpson diversity index is 0.98, 0.97, and 0.94, and the average value of the fundamental biodiversity number is 50.35, 48.46, and 42.74, for the *q_i_*–metacommunity with σ = 0.1, 1, and 4 respectively. Reduced biodiversity and evenness in the *q_i_*–metacommunity directly result in lower fundamental biodiversity numbers of the local communities when σ is large (Table 1). Furthermore, species with relatively high dispersal abilities can result in very high abundance in the local communities (Fig. 1, 2), causing the estimated *m* increases with increasing σ.

However, the effects of migration asymmetry on local community structures are moderate compared with those of asymmetries in fecundity or mortality rates. In real communities, both mortality rates and dispersal distances can vary considerably among species. For example, in 1990–1995, the mortality rates of the tree species in Barro Colorado Island (BCI) rainforest varied from 0.44 to 16.4% per year for the 63 species with over 50 individuals ≥10 cm dbh [29]. Estimated modal dispersal distances of seeds in temperate and tropical forests vary from about 1 to 40 m [30]. For comparison, we simulated the effects of asymmetry in mortality rates using the method similar to that used by Zhou and Zhang [16]. However, we assumed that each species per capita mortality factor was drawn from the log-normal distribution with mean = 1 and standard deviation = σ rather than assuming fecundity asymmetry. We found that a standard deviation in species per capita mortality factor of 0.01 (1% compared with the mean of 1) had profound influences on the community structure and removed about 50% or more of the species from the metacommunity or local communities (results not shown). Similar results were found when species differed slightly in their per capita fecundity rates [16]. But a standard deviation in species regional dispersal ability of 0.1, which was 10% of the mean, had almost no effect on the community structure (Fig. 1). We also found that a local neutral community with asymmetric regional dispersal can preserve the neutral pattern unless very large asymmetry in migration is assumed. However, this does not necessarily mean that dispersal asymmetry is not important or that the neutral assumption with respect to migration is verified.

On the one hand, large differences among species migration probability could have significant effects on local community structures (Fig.1). Extremely asymmetric dispersals result in decreased species richness and different species abundance distributions compared with predictions by the neutral model. Considering the effects of species differences on community structures, Hubbell argued that the neutral assumption can act well once species fitness are equalized, i.e. by trade-off between seed size vs. number and fecundity vs. death rate [9]. Also one study regarded such trade-offs as a bridge to reconcile the neutral theory and species difference [31]. However, as Turnbull et al. [32] suggested trade-offs are not always neutral. Turnbull et al. showed that even when the trade-off between seed size vs. number is equalizing, random variations in the initial number of seeds colonizing a site can generate an advantage to small-seeded species and result in deterministic competitive exclusion [32]. More investigations are needed before we can understand the relative roles of niches, species differences and neutrality in structuring ecological communities.

On the other hand, more realistic dispersal processes and dispersal asymmetries may produce different conclusions compared with what we have reported here. In this paper, the migration process is modeled as a spatially implicit process from the metacommunity to the local community, which is the same as spatially implicit neutral models did [6], [9], [13], [15], [33]. This simplification should be valid in some cases. In real communities, however, migration is a relatively “local” process. For instance, dispersal of seeds of forest trees is generally confined within a short distance [34]. In another study, the estimations of modal dispersal distances of seeds in temperate and tropical forests are generally around 10 m [30]. Hence, spatially explicit migration should be considered in relevant models for a better understanding of the consequences of symmetric and asymmetric dispersals [35].

In this study, we relaxed the strict symmetry assumptions of the neutral model by incorporating differences in species dispersal rate from the metacommunity into local communities. As in the neutral model, diversity in the local community is maintained as a dynamic equilibrium between the extinction of resident species and immigration of new species from the metacommunity [25], [36], [37]. Neutral local communities with asymmetric immigration end up with lower species richness and species abundance distributions that can be quite different from those produced by the unified neutral theory. This is consistent with the conclusion made by Hu et al. [26]. The community dynamics is governed by a random drift in both Hu et al. [26] and in this paper. However, the variances in migration and the corresponding consequences are different. Hu et al. actually modeled the stochastic variance in migration among species and in the total number of migrants [26]. The variances reduced the species richness mainly through the loss of rare species. But with the asymmetric dispersal introduced in this paper, local community dynamics become deterministic as species with higher dispersal abilities have higher relative abundances in the local communities (Fig. 2). Thus, rare species are those with less probabilities of immigration from the metacommunity. Such species are less likely to enter local communities. Furthermore, once these species go extinct, it is less possible for them to immigrate from the metacommunity because of their lower immigration rates. This is consistent with some empirical observations. For instance, Hovestadt et al. [38] investigated the woody plant species composition of 49 forest islands, 18 savanna, and 3 gallery forest plots in the Comoé National Park (Ivory Coast). They found that the species composition of these forest islands was to some extent determined by species seed dispersal abilities, with those species lacking in long distance seed dispersal mechanisms being correspondingly rare in forest islands. In another study, Burns evaluated the relationship between seed dispersal and plant community structures on islands off the coast of British Columbia, Canada [39]. He found that island plant communities were dominated by fleshy-fruited species rather than dry-fruited species. Patterns in seed dispersal were consistent with the differences in diversity. Birds dispersed thousands of fleshy-fruited seeds out to islands, while mainland-island dispersal of dry-fruited species was not possible.

Based on the results of our simulation and those achieved by Zhou and Zhang [16], species abundance distributions can deviate considerably from those reproduced by the neutral model, given that species differ in their vital demographic parameters. However, species abundance distributions accounting for species differences can often be well fitted by the neutral model [16], [18], [40]. This demonstrates the strong ability of the neutral theory in predicting species abundance distributions in both neutral and non-neutral communities. On the other hand, the neutral theory not only fails to measure species differences in non-neutral communities and neutral communities with asymmetric immigration but it can neither predict vital parameter values such as the fundamental biodiversity number[16], [18]. Hence, the ability of the curve-fitting measure is rather limited. Species traits may be important in explaining species abundance distribution and underlying mechanisms.

One vital weakness of the neutral model is that it cannot provide any information about species composition and the relationship between species richness and the ecosystem function, which must be answered by community ecologists. Once species differences are introduced into the neutral model, species can also dynamically coexist despite the asymmetry in competitive ability or migration and positive relationships between the species competition ability or migration rate and its abundance emerge [16]. In this sense, a nearly neutral model incorporating slight species differences in demographic rates may be more useful than a purely neutral model.

## Methods

We constructed a neutral metacommunity of size *J_m_* and with the fundamental biodiversity number *θ* following the algorithm described by Hubbell [9] (Page 291). The fundamental biodiversity number is a measurement of biodiversity and equals twice the product of the community size and the speciation rate. As in all of the spatially implicit neutral models, dispersal limitation was assumed to occur only from the metacommunity to a local community [9], [13]. The metacommunity itself was neutral except the difference in species dispersal rate from the metacommunity into the local community. In doing so, we selected each species' per capita dispersal factor into the local community from log-normal distribution with mean = 1, and standard deviation  = σ. Other distributions (i.e. normal or uniform) led to essentially the same conclusion (results not shown). We denoted by *m_i_* the per capita dispersal factor of species *i* into a local community. σ = 0 is the neutral case with all *m_i_* being equal to1.

With the background metacommunity formed as described above, we explored the effect of asymmetric dispersal limitation from the metacommunity to the local community on species richness and species abundance distributions in the local community. We sampled local communities of size *J*, with the Hubbell's migration parameter equaling *m*. Contrasted with the neutral model, the initial relative abundance of species *i* in the local community is proportional to *m_i_P_i_*, where *P_i_* is the abundance of species *i* in the corresponding metacommunity. The dynamics of the local community were determined by local birth, death and immigration from the metacommunity as described below.

To model the dynamics of a local community, we first randomly eliminated an individual from the local community. With the probability *m* (migration coefficient) the vacant site was occupied by an immigrant, and the immigrant belonged to species *i* with probability 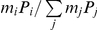. Otherwise an offspring of an existing individual in the local community replaced the dead and, with the probability 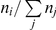 the new recruit came from species *i*, where *n_i_* is the abundance of species *i* in the local community. The local dynamics were iterated for 20 000 turnovers of the local community, which was long enough for the community to reach a stochastic equilibrium.

In the simulations, we assumed that *J_m_* = 1×10^6^, *θ* = 50, *J* = 1×10^4^, *m* = 0.01, 0.1 and 0.3 respectively. For each value of *m*, we selected the value of standard deviation of species per capita dispersal factor σ to be 0 (neutral case), 0.1, 1, and 4 respectively. The values of *m* were similar to that estimated for tropical rainforest [13]. For each parameter set, the distributions of species abundances were the average of 100 replicate local communities drawn from the same metacommunity, assuming that the mean migration rate *m* and the standard deviation *σ* were the same. Additionally, while calculating the distribution of species' abundance in the local community, we recorded the relationship between species' per capita dispersal factors and their relative abundances in the local community at equilibrium. Then we fitted the simulated communities with Hubbell's neutral model and tested the goodness of fit as follows. For each simulated abundance data set, we estimated the fundamental biodiversity number, migration rate and the log-likelihood value (LV) using the method proposed by Etienne [6]. Then we constructed 100 neutral communities using the estimated parameter values. Fitting these communities by the neutral model provided 100 log-likelihood values (LV*_i_*, *i* = 1, …, 100). We obtained a percentage value by comparing the LV and LV*_i_* (*i* = 1, …, 100). A percentage value that is around or larger than 0.5 means the simulated abundance data set can be well fitted by the neutral model [41].
